# Bittersweet: infective complications of drug-induced glycosuria in patients with diabetes mellitus on SGLT2-inhibitors: two case reports

**DOI:** 10.1186/s12879-021-05982-3

**Published:** 2021-03-20

**Authors:** Caroline Bartolo, Victoria Hall, N. Deborah Friedman, Chloe Lanyon, Andrew Fuller, C. Orla Morrissey, Eugene Athan

**Affiliations:** 1 Department of Infectious Diseases, Barwon Health, Geelong, Victoria Australia; 2 Department of Infectious Diseases, Alfred Hospital, Melbourne, Victoria Australia; 3 Monash University, Melbourne, Victoria Australia; 4 Deakin University, Melbourne, Victoria Australia

**Keywords:** Candidemia, SGLT2 inhibitor, Glycosuria, Case report, Genital mycotic infection, Prostatic abscess

## Abstract

**Background:**

Sodium-glucose co-transporter 2 (SGLT2) inhibitors are novel hypoglycemic agents which reduce reabsorption of glucose at the renal proximal tubule, resulting in significant glycosuria and increased risk of genital mycotic infections (GMI). These infections are typically not severe as reported in large systematic reviews and meta-analyses of the medications. These reviews have also demonstrated significant cardiovascular benefits through other mechanisms of action, making them attractive options for the management of Type 2 diabetes mellitus (T2DM). We present two cases with underlying abnormalities of the urogenital tract in which the GMI were complicated and necessitated cessation of the SGLT2 inhibitor.

**Case presentations:**

Both cases are patients with T2DM on empagliflozin, an SGLT2 inhibitor. The first case is a 64 year old man with *Candida albicans* balanitis and candidemia who was found to have an obstructing renal calculus and prostatic abscess requiring operative management. The second case describes a 72 year old man with *Candida glabrata* candidemia who was found to have prostatomegaly, balanitis xerotica obliterans with significant urethral stricture and bladder diverticulae. His treatment was more complex due to fluconazole resistance and concerns about urinary tract penetration of other antifungals. Both patients recovered following prolonged courses of antifungal therapy and in both cases the SGLT2 inhibitor was ceased.

**Conclusions:**

Despite their cardiovascular benefits, SGLT2 inhibitors can be associated with complicated fungal infections including candidemia and patients with anatomical abnormalities of the urogenital tract may be more susceptible to these infections as demonstrated in these cases. Clinicians should be aware of their mechanism of action and associated risk of infection and prior to prescription, assessment of urogenital anatomical abnormalities should be performed to identify patients who may be at risk of complicated infection.

## Background

Sodium-glucose cotransporter 2 (SGLT2) inhibitors are novel oral hypoglycemic agents which inhibit the re-uptake of filtered sodium and glucose at the proximal convoluted tubule. Large drug efficacy trials have found substantial improvement in glycemic control, weight loss, a reduction in blood pressure and cardiovascular mortality; so, are an attractive option for the management of Type 2 diabetes mellitus (T2DM) [1–6].

SGLT2 inhibitors cause significant glycosuria, associated with a nearly five-fold increased risk of genital mycotic infections (GMI; balanitis in men and vulvovaginitis in women) [4]. We describe two cases of candidemia secondary to GMI in patients on SGLT2 inhibitors, which led to significant morbidity and the subsequent discontinuation of the SGLT2 inhibitor.

## Case presentations

A 64-year-old man with T2DM, managed with empagliflozin 10mg daily, which had been commenced 2 years prior, metformin and sitagliptin, presented to the emergency department with progressive lethargy, fevers and dysuria. The symptoms had progressed despite receiving 5 days of oral cephalexin a few weeks prior following collection of a urine sample which grew *Candida albicans* alone. On examination, he was febrile (39.6°C), tachycardic (108 beats per minute) and hypotensive (87/55 mmHg) and blood sugar level (BSL) was measured at 39mmol/L (702mg/dL). Examination revealed balanoposthitis but was otherwise unremarkable.

Initial investigations showed significantly raised inflammatory markers: C-reactive protein (CRP) of 287mg/L, total white blood cell count (WCC) of 32.1 × 10^9^/L with a predominant neutrophilia of 28.2 × 10^9^/L and acute kidney injury with serum creatinine 208μmol/L (CrCl 28ml/min). HbA1C was 11.0% (< 5.7%) and prostate specific antigen (PSA) 2.76mcg/L (< 4.51mcg/L).

He was transferred to the intensive care unit (ICU) for inotropic support; 24 h into his admission a yeast was identified from two sets of blood cultures and he was commenced on intravenous (IV) anidulafungin. *Candida albicans* was subsequently isolated from blood, urine and urethral swab cultures. The minimum inhibitory concentration (MIC) for fluconazole was < 0.05μg/L (susceptible) and he was changed to IV fluconazole 400mg daily.

An abdomino-pelvic computed tomography scan demonstrated right-sided emphysematous pyelonephritis, a 7mm non-obstructing calculus in the right renal pelvis, a large (57x43x65mm) bladder diverticulum and emphysematous prostatitis. As he remained candidemic, repeat imaging on day 7 demonstrated gas in the prostate and that the renal calculus had migrated to the proximal ureter causing acute obstruction necessitating the urgent insertion of a nephrostomy tube. On day 12 he underwent anterograde ureteric stent insertion. A transperineal ultrasound on day 13 confirmed the presence of a prostatic abscess requiring cystoscopy, transurethral drainage of the prostatic abscess and transurethral resection of the prostate. Culture of the abscess grew *C. albicans* (fluconazole MIC < 0.05μg/L). Transesophaegeal echocardiography and ophthalmological examination were normal. The SGLT2 inhibitor was ceased and he was discharged home on oral fluconazole 400mg daily with an indwelling urinary catheter (IDC).

Three weeks later whilst still on fluconazole, he had an episode of fever and right flank pain. The renal calculus, stent and catheter were removed. He received a total of 6 weeks of fluconazole and recovered.

A 72-year-old man presented emergently to hospital after having driven his car into a concrete barrier at low speed. He was febrile and confused. He had a history of T2DM, managed with metformin and empagliflozin 10mg daily; the latter prescribed only 2 months prior. Examination did not reveal any injury sustained from the collision; however, it was significant for balanitis with a tender prostate and he was in acute urinary retention. BSL was significantly raised at 22.2mmol/L (400mg/dL).

Investigations revealed a CRP of 261mg/L, total WCC of 14.4 × 10^9^/L with a neutrophilia (13.4 × 10^9^/L), acute kidney injury with serum creatinine 113μmol/L (CrCl 55mL/min). HbA1C was 9.0% (< 5.7%) and PSA was 8.8mcg/L (< 4.51mcg/L). Blood cultures grew a yeast at 36 h and the patient was commenced empirically on IV caspofungin. The yeast was identified as *Candida glabrata*; urine and urethral swab specimens were also culture positive for *C. glabrata*. For all isolates the same susceptibility profile was observed, with a fluconazole MIC of 128μg/L (resistant). Susceptibility was confirmed to caspofungin (MIC 0.06μg/L), 5- flucytosine (MIC ≤0.06μg/L) and amphotericin B (MIC 0.5μg/L). Blood culture clearance occurred after 48 h of caspofungin.

A renal tract ultrasound demonstrated prostatic enlargement, bladder diverticulae and an elevated post-void residual volume (>300mls). On day 5 of his admission, he underwent rigid cystoscopy and urethral dilatation with the significant findings of a pinpoint meatal urethral stricture, glandular adhesions and balanitis xerotica obliterans. During the procedure an IDC was inserted to relieve the bladder outlet obstruction. Transthoracic echocardiogram and ophthalmological examination were normal.

Caspofungin was changed to micafungin 100mg IV daily. Fluconazole was added and the dose was reduced from 1200mg (10mg/kg) to 800mg daily due to QTc prolongation. 5-flucytosine 25mg/kg QID was added. He received a total of 4 weeks treatment with clinical and microbiological cure. The SGLT2 inhibitor was ceased.

## Discussion & Conclusions

SGLT2 inhibitors are selective membrane transport inhibitors which act on the proximal convoluted tubule and prevent glucose and sodium absorption back into the circulation resulting in glycosuria. In a randomised, placebo-controlled trial in patients taking varying doses of empagliflozin, urinary glucose excretion reached up to 90g/day [1]. The average daily volume of urine excreted by participants was 3 l equating to a urinary glucose concentration of 30g/L, roughly equivalent to that of Sabouraud agar, the preferred growth medium for optimal yeast culture including *Candida species* providing a biologically plausible explanation for the observed increased risk of GMI.

Patients with T2DM are more likely to have genital tract colonisation with *Candida spp* [7] and the risk of GMI is higher in patients with diabetes. In a population-based study, in the UK, diabetic women had a risk ratio of 1.81 (95% CI 1.64–2.0) for vaginitis and men had a risk ratio of 2.85 (2.84–4) for balanitis compared with those without diabetes [7]. A number of systematic reviews and meta-analyses, to date, have shown a 4- to 8-fold increased risk of GMI in patients taking SGLT2 inhibitors compared with placebo or other hypoglycemic agents [2–6, 8]. Mechanisms for this increased risk include increased adherence of *Candida* to the genital tract and reduced host immune response in patients with T2DM [9]. This, combined with the further increase in glycosuria in patients on SGLT2 inhibitor therapy and pre-existing anatomical abnormalities of the urogenital tract may predispose to complicated urinary tract infection (UTI).

The U.S. Food and Drug Administration (FDA) issued a warning about the risk of complicated UTI in patients taking SGLT2 inhibitors in 2015 after 19 cases of complicated UTI were reported from March 2013 through October 2014. In contrast, a large population-based cohort study found no increased risk of serious UTI in patients taking an SGLT2 inhibitor compared with other hypoglycemic agents. However, this study excluded patients with higher risk of UTI (including those with anatomical abnormalities such as prostatomegaly and obstructive defects of the renal pelvis and ureter including calculi) [10]. In patients with a higher predisposition for severe UTI, complications can include candidemia which is associated with up to 47% mortality, endophthalmitis and endocarditis [11].

Duration of therapy for fungal prostatic abscess and candidemia from a urinary tract source are not well-defined. These two cases were treated for 4–6 weeks. Case 1 had prolonged candidemia until source control (abscess drainage) was achieved. It has been reported that successful treatment of *Candida* prostatitis has been achieved with micafungin when paired with surgical drainage [12]. Case 2 highlights the potential challenges in treating fluconazole-resistant *Candida spp* infection from a urinary tract source.

Fluconazole is the usual drug of choice for *Candida* UTI [11, 13, 14]. It is mostly eliminated unchanged (80%) in the urine resulting in urinary concentrations that are 10–20 times that of plasma (levels >100μg/ml) [11, 13, 14]. Therefore, urinary concentrations are able to exceed MIC for susceptible but dose dependent (MIC 16-32μg/mL) and even resistant (MIC >64μg/mL) isolates. Higher dosing of fluconazole (12mg/kg) is often recommended for the treatment of susceptible *C. glabrata* infections; however, there are limited clinical data to support this advice [14–16]. Other azoles (e.g. posaconazole, voriconazole) are minimally excreted in the urine and therefore not recommended [11, 14]. Flucytosine has broad anti-fungal activity and excellent oral bioavailability, is mostly excreted unchanged in urine (97%) and similarly achieves high urinary concentrations (~ 60 times that of plasma) [11, 14, 16]. It is usually given in combination as resistance can develop when it is used as monotherapy; due to its short half-life it is dosed at 25mg/kg 4 times daily and therapeutic drug monitoring is recommended (as in case 2) [11, 14, 16]. There is emerging evidence that echinocandins, particularly micafungin, can achieve adequate clinical efficacy when correlated with PK/PD ratios [17]; and in the treatment of candidemia from a urinary tract source, similar outcomes have been achieved with echinocandins compared with fluconazole [18]. Case 2 was thus treated with a combination of high dose fluconazole plus flucytosine for optimal penetration into the urinary tract, with the addition of micafungin due to the severity of the infection.

SGLT2 inhibitors are prescribed more frequently in T2DM due to their favorable cardiovascular outcomes. However, as demonstrated in these cases, there appears to be an associated increased risk of complicated fungal infection in patients with underlying abnormalities of the urogenital tract. We suggest that prior to the prescription of SGLT2 inhibitors, urogenital abnormalities should be identified and treated. The risks and benefits of these medications should be discussed with the patient and alternate hypoglycemic agents considered if the risk of GMI is considered to outweigh the benefits.

## Data Availability

Not applicable.

## Abbreviations

SGLT2: Sodium-glucose co-transporter 2
GMI: Genital mycotic infections
T2DM: Type 2 diabetes mellitus
BSL: Blood sugar level
CRP: C-reactive protein
WCC: White blood cell count
CrCl: Creatinine clearance
HbA1c: Glycated haemoglobin
PSA: Prostate specific antigen
ICU: Intensive care unit
IV: Intravenous
MIC: Minimum inhibitory concentration
IDC: Indwelling catheter
QTc: Corrected QT interval
UTI: Urinary tract infection
